# Supplementary material for: Multi‐dataset identification of innovative feature genes and molecular mechanisms in keratoconus
Source: J Cell Mol Med. 2024 Sep 19;28(18):e70079. doi: 10.1111/jcmm.70079 (PMC11412914; doi:10.1111/jcmm.70079)
Supplement: Supplementary file 2 — Material S2. [file JCMM-28-e70079-s002.zip › 7.GSEA analysis/3.DBNDD1/KEGG_HUNTINGTONS_DISEASE.html]

Details for gene set KEGG\_HUNTINGTONS\_DISEASE[GSEA]

|  || Dataset | DBNDD1.group.cls#h\_versus\_l |
| Phenotype | group.cls#h\_versus\_l |
| Upregulated in class | l |
| GeneSet | KEGG\_HUNTINGTONS\_DISEASE |
| Enrichment Score (ES) | -0.2672543 |
| Normalized Enrichment Score (NES) | -1.4810331 |
| Nominal p-value | 0.05487805 |
| FDR q-value | 0.65935016 |
| FWER p-Value | 0.82 |
Table: GSEA Results Summary

  

Fig 1: Enrichment plot: KEGG\_HUNTINGTONS\_DISEASE      
 Profile of the Running ES Score & Positions of GeneSet Members on the Rank Ordered List

  

| SYMBOL | TITLE | RANK IN GENE LIST | RANK METRIC SCORE | RUNNING ES | CORE ENRICHMENT || 1 | SLC25A31 | na | 2127 | 0.291 | -0.0329 | No |
| 2 | DNAI1 | na | 2200 | 0.288 | -0.0067 | No |
| 3 | GRM5 | na | 2394 | 0.277 | 0.0150 | No |
| 4 | PPARGC1A | na | 2929 | 0.255 | 0.0246 | No |
| 5 | DNAI2 | na | 3800 | 0.220 | 0.0211 | No |
| 6 | TBPL2 | na | 4062 | 0.212 | 0.0343 | No |
| 7 | CLTC | na | 6408 | 0.139 | -0.0198 | No |
| 8 | HTT | na | 7851 | 0.106 | -0.0511 | No |
| 9 | APAF1 | na | 8227 | 0.099 | -0.0521 | No |
| 10 | POLR2J2 | na | 8504 | 0.094 | -0.0508 | No |
| 11 | COX7A2L | na | 8857 | 0.088 | -0.0523 | No |
| 12 | COX6A2 | na | 8938 | 0.087 | -0.0461 | No |
| 13 | PLCB2 | na | 9009 | 0.086 | -0.0397 | No |
| 14 | COX4I2 | na | 9035 | 0.085 | -0.0321 | No |
| 15 | CREBBP | na | 9242 | 0.082 | -0.0300 | No |
| 16 | CASP8 | na | 9432 | 0.079 | -0.0277 | No |
| 17 | DNALI1 | na | 9791 | 0.074 | -0.0307 | No |
| 18 | AP2A2 | na | 10114 | 0.070 | -0.0331 | No |
| 19 | COX6B2 | na | 10294 | 0.067 | -0.0317 | No |
| 20 | NDUFB5 | na | 10463 | 0.065 | -0.0302 | No |
| 21 | UCP1 | na | 10632 | 0.063 | -0.0289 | No |
| 22 | NDUFC2 | na | 11325 | 0.054 | -0.0435 | No |
| 23 | NDUFV3 | na | 11702 | 0.050 | -0.0495 | No |
| 24 | GPX1 | na | 11797 | 0.049 | -0.0474 | No |
| 25 | CREB3 | na | 12277 | 0.045 | -0.0568 | No |
| 26 | NDUFAB1 | na | 12930 | 0.039 | -0.0719 | No |
| 27 | NDUFB1 | na | 13049 | 0.038 | -0.0716 | No |
| 28 | NDUFS6 | na | 13068 | 0.038 | -0.0684 | No |
| 29 | CASP3 | na | 13339 | 0.035 | -0.0727 | No |
| 30 | NDUFB10 | na | 13393 | 0.035 | -0.0708 | No |
| 31 | UQCR11 | na | 13449 | 0.035 | -0.0690 | No |
| 32 | BAX | na | 13486 | 0.034 | -0.0666 | No |
| 33 | NDUFB8 | na | 13532 | 0.034 | -0.0646 | No |
| 34 | NDUFB7 | na | 13785 | 0.032 | -0.0688 | No |
| 35 | POLR2H | na | 13862 | 0.031 | -0.0679 | No |
| 36 | COX8A | na | 13890 | 0.031 | -0.0657 | No |
| 37 | TP53 | na | 13962 | 0.030 | -0.0648 | No |
| 38 | TFAM | na | 14010 | 0.030 | -0.0632 | No |
| 39 | POLR2C | na | 14050 | 0.030 | -0.0614 | No |
| 40 | SDHC | na | 14109 | 0.029 | -0.0602 | No |
| 41 | POLR2I | na | 14158 | 0.029 | -0.0588 | No |
| 42 | CLTA | na | 14326 | 0.028 | -0.0609 | No |
| 43 | NDUFA9 | na | 14400 | 0.027 | -0.0603 | No |
| 44 | HDAC1 | na | 14495 | 0.026 | -0.0604 | No |
| 45 | NDUFA4L2 | na | 14523 | 0.026 | -0.0586 | No |
| 46 | NDUFS1 | na | 14531 | 0.026 | -0.0563 | No |
| 47 | SLC25A6 | na | 14804 | 0.024 | -0.0617 | No |
| 48 | NDUFS4 | na | 14861 | 0.024 | -0.0610 | No |
| 49 | POLR2J3 | na | 14921 | 0.023 | -0.0604 | No |
| 50 | NDUFA10 | na | 14952 | 0.023 | -0.0590 | No |
| 51 | NDUFS2 | na | 15047 | 0.022 | -0.0595 | No |
| 52 | PPID | na | 15089 | 0.022 | -0.0585 | No |
| 53 | SLC25A5 | na | 15264 | 0.021 | -0.0615 | No |
| 54 | COX6B1 | na | 15292 | 0.021 | -0.0602 | No |
| 55 | COX5B | na | 15394 | 0.020 | -0.0612 | No |
| 56 | POLR2J | na | 15425 | 0.020 | -0.0601 | No |
| 57 | COX7B2 | na | 15452 | 0.020 | -0.0589 | No |
| 58 | COX7B | na | 15536 | 0.019 | -0.0595 | No |
| 59 | NDUFS8 | na | 15627 | 0.018 | -0.0603 | No |
| 60 | RCOR1 | na | 15706 | 0.018 | -0.0608 | No |
| 61 | COX7A2 | na | 15716 | 0.018 | -0.0593 | No |
| 62 | NDUFA1 | na | 15785 | 0.017 | -0.0596 | No |
| 63 | CREB3L1 | na | 15797 | 0.017 | -0.0582 | No |
| 64 | NDUFC1 | na | 15837 | 0.017 | -0.0577 | No |
| 65 | NDUFA7 | na | 15872 | 0.017 | -0.0570 | No |
| 66 | DCTN2 | na | 15998 | 0.016 | -0.0590 | No |
| 67 | UQCR10 | na | 16010 | 0.016 | -0.0578 | No |
| 68 | NDUFB3 | na | 16011 | 0.016 | -0.0563 | No |
| 69 | POLR2D | na | 16178 | 0.015 | -0.0596 | No |
| 70 | SDHD | na | 16304 | 0.014 | -0.0619 | No |
| 71 | NDUFA8 | na | 16485 | 0.013 | -0.0658 | No |
| 72 | NDUFB2 | na | 16493 | 0.013 | -0.0648 | No |
| 73 | SP1 | na | 16584 | 0.012 | -0.0662 | No |
| 74 | UQCRC1 | na | 16639 | 0.012 | -0.0666 | No |
| 75 | NDUFS3 | na | 16717 | 0.011 | -0.0678 | No |
| 76 | UQCRC2 | na | 16785 | 0.011 | -0.0686 | No |
| 77 | COX4I1 | na | 16804 | 0.011 | -0.0681 | No |
| 78 | POLR2K | na | 17057 | 0.009 | -0.0745 | No |
| 79 | COX7C | na | 17085 | 0.009 | -0.0744 | No |
| 80 | NDUFS7 | na | 17112 | 0.009 | -0.0743 | No |
| 81 | NDUFA4 | na | 17126 | 0.009 | -0.0739 | No |
| 82 | NDUFB9 | na | 17132 | 0.009 | -0.0732 | No |
| 83 | UQCRQ | na | 17160 | 0.008 | -0.0731 | No |
| 84 | COX6C | na | 17183 | 0.008 | -0.0729 | No |
| 85 | TAF4 | na | 17201 | 0.008 | -0.0726 | No |
| 86 | NDUFA3 | na | 17211 | 0.008 | -0.0721 | No |
| 87 | DCTN4 | na | 17230 | 0.008 | -0.0719 | No |
| 88 | VDAC3 | na | 17362 | 0.007 | -0.0749 | No |
| 89 | AP2M1 | na | 17638 | 0.005 | -0.0824 | No |
| 90 | NDUFA5 | na | 17695 | 0.005 | -0.0835 | No |
| 91 | COX5A | na | 17706 | 0.005 | -0.0833 | No |
| 92 | CYC1 | na | 17708 | 0.005 | -0.0829 | No |
| 93 | DNAL4 | na | 17756 | 0.005 | -0.0838 | No |
| 94 | NDUFV1 | na | 17902 | 0.003 | -0.0876 | No |
| 95 | REST | na | 17910 | 0.003 | -0.0875 | No |
| 96 | POLR2A | na | 17989 | 0.003 | -0.0895 | No |
| 97 | POLR2G | na | 18012 | 0.003 | -0.0899 | No |
| 98 | NDUFV2 | na | 18101 | 0.002 | -0.0922 | No |
| 99 | UQCRH | na | 18129 | 0.002 | -0.0928 | No |
| 100 | SDHB | na | 18300 | 0.001 | -0.0977 | No |
| 101 | NDUFS5 | na | 18515 | -0.001 | -0.1038 | No |
| 102 | SLC25A4 | na | 18531 | -0.001 | -0.1041 | No |
| 103 | POLR2B | na | 18643 | -0.002 | -0.1071 | No |
| 104 | NDUFA2 | na | 18897 | -0.004 | -0.1141 | No |
| 105 | COX6A1 | na | 18979 | -0.004 | -0.1160 | No |
| 106 | AP2S1 | na | 18992 | -0.004 | -0.1159 | No |
| 107 | POLR2L | na | 19025 | -0.005 | -0.1164 | No |
| 108 | NDUFB4 | na | 19246 | -0.006 | -0.1221 | No |
| 109 | ITPR1 | na | 19252 | -0.006 | -0.1216 | No |
| 110 | UQCRFS1 | na | 19283 | -0.007 | -0.1218 | No |
| 111 | UQCRB | na | 19342 | -0.007 | -0.1228 | No |
| 112 | TBPL1 | na | 19474 | -0.008 | -0.1258 | No |
| 113 | PLCB3 | na | 19605 | -0.010 | -0.1286 | No |
| 114 | PLCB4 | na | 19707 | -0.010 | -0.1305 | No |
| 115 | CASP9 | na | 19881 | -0.012 | -0.1343 | No |
| 116 | TBP | na | 20043 | -0.013 | -0.1376 | No |
| 117 | CYCS | na | 20083 | -0.014 | -0.1374 | No |
| 118 | POLR2E | na | 20180 | -0.015 | -0.1388 | No |
| 119 | NDUFA6 | na | 20241 | -0.015 | -0.1390 | No |
| 120 | CREB3L3 | na | 20550 | -0.018 | -0.1462 | No |
| 121 | SOD1 | na | 20880 | -0.021 | -0.1536 | No |
| 122 | VDAC2 | na | 21354 | -0.025 | -0.1648 | No |
| 123 | COX7A1 | na | 21665 | -0.029 | -0.1710 | No |
| 124 | GNAQ | na | 21825 | -0.030 | -0.1726 | No |
| 125 | NDUFB6 | na | 21907 | -0.031 | -0.1719 | No |
| 126 | DCTN1 | na | 22013 | -0.032 | -0.1717 | No |
| 127 | SDHA | na | 22031 | -0.032 | -0.1691 | No |
| 128 | AP2B1 | na | 22449 | -0.037 | -0.1775 | No |
| 129 | CREB3L4 | na | 22477 | -0.037 | -0.1747 | No |
| 130 | VDAC1 | na | 22895 | -0.042 | -0.1826 | No |
| 131 | POLR2F | na | 23137 | -0.045 | -0.1852 | No |
| 132 | CREB3L2 | na | 23589 | -0.051 | -0.1932 | No |
| 133 | AP2A1 | na | 23676 | -0.052 | -0.1906 | No |
| 134 | CLTB | na | 23684 | -0.052 | -0.1857 | No |
| 135 | HDAC2 | na | 24190 | -0.059 | -0.1945 | No |
| 136 | EP300 | na | 24292 | -0.060 | -0.1915 | No |
| 137 | CREB1 | na | 24296 | -0.060 | -0.1856 | No |
| 138 | DNAH1 | na | 24452 | -0.062 | -0.1840 | No |
| 139 | SIN3A | na | 25327 | -0.076 | -0.2018 | No |
| 140 | SOD2 | na | 25515 | -0.079 | -0.1995 | No |
| 141 | HAP1 | na | 26530 | -0.098 | -0.2191 | No |
| 142 | DLG4 | na | 26655 | -0.101 | -0.2128 | No |
| 143 | GRIN2B | na | 26883 | -0.105 | -0.2090 | No |
| 144 | DNAL1 | na | 27047 | -0.109 | -0.2030 | No |
| 145 | GRIN1 | na | 27251 | -0.114 | -0.1977 | No |
| 146 | COX8C | na | 27951 | -0.130 | -0.2052 | No |
| 147 | BDNF | na | 28409 | -0.141 | -0.2045 | No |
| 148 | NRF1 | na | 29611 | -0.176 | -0.2219 | No |
| 149 | BBC3 | na | 29642 | -0.177 | -0.2054 | No |
| 150 | HIP1 | na | 31781 | -0.253 | -0.2424 | Yes |
| 151 | DNAH2 | na | 31866 | -0.257 | -0.2195 | Yes |
| 152 | TAF4B | na | 32013 | -0.264 | -0.1978 | Yes |
| 153 | PLCB1 | na | 32023 | -0.265 | -0.1720 | Yes |
| 154 | PPARG | na | 32783 | -0.313 | -0.1632 | Yes |
| 155 | CLTCL1 | na | 33772 | -0.401 | -0.1524 | Yes |
| 156 | TGM2 | na | 34499 | -0.551 | -0.1191 | Yes |
| 157 | CREB5 | na | 34560 | -0.587 | -0.0631 | Yes |
| 158 | IFT57 | na | 34658 | -0.690 | 0.0020 | Yes |
Table: GSEA details [plain text format]

  

Fig 2: KEGG\_HUNTINGTONS\_DISEASE      
 Blue-Pink O' Gram in the Space of the Analyzed GeneSet

  

Fig 3: KEGG\_HUNTINGTONS\_DISEASE: Random ES distribution      
 Gene set null distribution of ES for **KEGG\_HUNTINGTONS\_DISEASE**
